# Young people’s perspectives on integrating physical activity interventions into youth substance use treatment practice: a mixed-methods study

**DOI:** 10.1186/s13722-025-00607-5

**Published:** 2025-10-09

**Authors:** Lisa Klamert, Melinda Craike, Gillinder Bedi, Susan Kidd, Alice Sweeting, Alexandra G. Parker

**Affiliations:** 1 Institute for Health and Sport, Victoria University, 70/104 Ballarat Rd, Footscray, VIC 3011 Australia; 2 Centre for Youth Mental Health, The University of Melbourne, Melbourne, VIC 3052 Australia; 3 Orygen, Melbourne, VIC 3052 Australia; 4 Mitchell Institute for Education and Health Policy, Victoria University, Melbourne, VIC 3011 Australia; 5 Acute Care Service, Tweed Byron Mental Health, Northern NSW Health District, Tweed Heads, Australia

**Keywords:** Adolescent, Young adult, Recreational drug use, Exercise, Surveys and questionnaires, Psychosocial intervention, Focus groups

## Abstract

**Background:**

Physical activity (PA) interventions may benefit youth with problematic substance use (SU); however, the acceptability of these interventions in young people is poorly understood. In this mixed-methods study, predictors and correlates of treatment acceptability of PA interventions as part of SU treatment were investigated, and young people’s perspectives on PA intervention (e.g., perceived barriers and service-related needs) were explored.

**Methods:**

Young people aged 16-25 years (n=145) with problematic SU completed a quantitative online survey on substance use, PA engagement, treatment acceptability, and perceived barriers and benefits of PA. Data were analysed using data mining and modelling approaches. Four participants aged 18-25 years participated in a subsequent, semi-structured focus group; data were analysed using qualitative content analysis. Quantitative and qualitative findings were integrated using an established model of behaviour change (COM-B).

**Results:**

Generalised additive modelling identified perceived PA barriers to be a predictor of treatment acceptability (*p*≤.001). Decision tree analyses confirmed that lower psychological distress (1^st^ partition, *p*<.001) and higher PA levels (2^nd^ partition, *p*=.03) predicted lower perceived PA barriers. Latent class analysis suggested a 2-class model differentiating young people at moderate substance-related risk, reporting low psychological distress and perceived PA barriers (class 1) from young people at severe substance-related risk, reporting higher psychological distress and perceived barriers. Qualitative findings revealed substantial barriers to PA, including substance-related, mental health, access, and social barriers. Together, findings illustrated complex interactions between different dimensions related to behaviour change and areas where clinical services may increase young people’s capability, opportunity and motivation to prompt behaviour change.

**Conclusions:**

PA levels and psychological distress predict perceived barriers to PA in young people with problematic SU. PA barriers predict treatment acceptability of PA interventions. Knowledge of such predictors may inform treatment decisions by clinicians. Young people’s insights should be integrated into PA intervention research to inform intervention and understand the unique barriers, preferences and needs of youth affected by problematic SU. Integration of young people’s perspectives may increase behaviour change, as well as motivation, engagement and positive feelings in young people participating in PA interventions within substance use treatment.

## Background

### Description of research problems/questions

Substance use in young people has overall declined in recent years [1, 2]; however, it remains a global health concern due to the many associated risks, such as disruption of important developmental trajectories [3–5]. Additionally, new and underexplored substance use patterns have emerged in recent years, such as an increase in e-cigarette use [6, 7].

Adolescence to young adulthood is the peak period of onset for many mental health disorders, including substance use disorders, with 48.8% of substance use disorders and approximately 75% of other mental health disorders emerging before the age of 25 years [8–10]. High levels of psychological distress, ineffective coping and comorbid problematic substance use are associated with long-term difficulties in education, relationships and well-being [11]. Despite a clear need for support, many young people continue to have limited access to clinical services (also referred to as treatment or mental health services) [12].

Other health behaviours that are compromised during adolescence include physical activity (PA). According to international guidelines, children and adolescents are recommended to engage in at least 60 min of moderate to vigorous intensive physical activity per day, young adults aged 18–25 years at least 150 min of moderate to vigorous intensity activity per week [13]. While only 19% of adolescents (11–17 years) were sufficiently active according to recommendations in 2016 [14], physical activity engagement in young adults has been neglected in research altogether [15].

PA has received growing recognition as a supplemental treatment to existing substance use and mental health interventions due to its many benefits for health and well-being [16, 17]. According to the WHO, physical activity refers to “*bodily movement produced by skeletal muscles that requires energy expenditure and includes all movement*,* including during leisure time*,* for transport to get to and from places*,* or as part of a person’s work or domestic activities*” [13]. Particularly in young people, physical activity may be suitable as an early intervention approach to reduce substance use [18], address mental health issues [19, 20], and increase PA engagement [21]. However, there remain several limitations, including the limited and heterogeneous evidence base around PA intervention for young people with substance use [18], the lack of understanding of experienced barriers to PA participation [22–24] and the acceptability of such interventions, particularly in young people with problematic substance use [25, 26].

Inclusion of young people in research and treatment design and listening to their values, preferences and experiences, is essential to developing evidence-based interventions [27], developing policies, and giving young people agency within relevant discussions and service planning [28, 29]. This is rarely done in research studies related to substance use [30]. Investigating the acceptability of integrated treatments that show promising treatment effects, such as physical activity, should therefore be a key focus to reduce problematic substance use and improve psychological and physical health in young people struggling with the complex challenges of problematic substance use and associated problems.

### Study aims

The study aimed to:

1. Explore acceptability of PA interventions, PA engagement, psychological distress, substance use and perceived PA barriers in young people aged 16–25 years with problematic substance use using a quantitative research survey.
2. Examine young people’s acceptability of PA interventions, perceived PA barriers, preferences and service-related needs regarding PA interventions using a qualitative focus group.
3. Identify areas of potential intervention to increase PA engagement (and potentially reduce SU), and develop recommendations to inform the integration of PA into substance use treatment based on integrated quantitative and qualitative findings.

## Methods

### Research design overview

A mixed methods approach using a sequential explanatory research design was applied to investigate young people’s perspectives on PA and the integration of PA interventions into substance use treatment. The study was aligned with the Capability, Opportunity, Motivation, and Behavior model (COM-B) model of behaviour [31].

Based on previous findings [18], an anonymous online survey was designed to assess young people’s substance use, physical activity engagement, perceived barriers and benefits to PA engagement and acceptability of tailored PA interventions as part of treatment. Survey participants aged 18 + were invited to participate in a subsequent focus group that investigated their perspectives regarding PA interventions in more detail. Eligibility criteria, sociodemographic information and current substance use were not re-assessed as part of the focus group as they had been captured anonymously during the previous survey. This approach mitigated potential legal and/or psychological risks associated with the capture of illicit (i.e., illegal) substance use data. Quantitative and qualitative phases were integrated during the interpretation and reporting stage. A comparison of stages is provided in Table 1.

**Table 1:** Implementation matrix of the mixed methods approach

	Quantitative	Qualitative	Mixed methods
Eligibility	• Aged 16–25 years • Problematic substance use • Previous and/or current engagement with clinical services, or stated willingness to engage with clinical services in the future (Single item)	• Aged 18–25 years • Problematic substance use • Previous and/or current engagement with clinical services, or stated willingness to engage with clinical services in the future (Single item)	Data from previous stages
Data type	Numeric data (quantitative)	Narrative/verbal data (qualitative)	Quantitative and qualitative data
Data source	Research survey	Focus group interview	Quantitative and qualitative data
Data analysis	Statistical analysis	Qualitative content analysis	Interpretative integration and discussion
Collected data	• Substance use (WHO ASSIST) • Sociodemographic information • PA engagement (AAS) • Perceived benefits to PA (EBBS subscale) • Perceived barriers to PA (EBBS subscale) • Psychological distress (K10) • Acceptability of tailored PA interventions (TAP)	• Previous experiences • PA intervention benefits • PA intervention barriers • Service-related needs (PA interventions) • Preferences (PA interventions)	Integrated quantitative and qualitative data

WHO ASSIST = The Alcohol, Smoking, and Substance Involvement Screening Test; AAS = Active Australia Survey; EBBS = Exercise Benefits and Barriers Scales; K10 = Kessler Psychological Distress Scale; TAP = Treatment Acceptability and Preferences measure

The research was approved by the Victoria University Human Research Committee, approval number HRE22-039. The manuscript was prepared in line with the APA Journal Article Reporting Standards for mixed methods (MMARS) and qualitative and quantitative research (JARS). A survey sample size of 50–100 individuals was pursued to achieve the relevant power requirements for regression and correlation analyses of the survey data [32].

## Participant recruitment

Survey participants (i.e., young people aged 16–25 years with problematic substance use) were recruited through university student services and digital social media platforms between July 2022 and September 2022. “Problematic substance use” was defined as being at moderate or severe risk of experiencing substance-related health issues as assessed with the WHO *Alcohol*,* Smoking*,* and Substance Involvement Screening Test* (ASSIST). Young people aged 16–17 years were invited to participate in the anonymous research survey based on the concept of mature minor [33–35], but were excluded from subsequent identifiable research. Maturity for survey participation was assessed at the start of the survey using a specific set of questions investigating if young people had understood the purpose, benefits and risks associated with the research [34, 35]. Participants aged 18 and older who completed the research survey were invited to express their interest in participating in the following focus group. Participants were screened according to eligibility as part of the survey. Data from quantitative and qualitative research stages were merged in the mixed-methods integration. Contrary to the commonly recommended focus group size of 8–12 people, a smaller group size was explicitly pursued as recommended for particular complex or sensitive topics [36]. Participants (Survey, focus group) were reimbursed non-cash gift vouchers.

## Data collection

The survey was implemented in Qualtrics, version May 2023 (SAP, Seattle, United States). It included validated questionnaires assessing substance use (WHO Alcohol, Smoking, and Substance Involvement Screening Test [ASSIST]) [37], cigarette dependence (Fagerstrom Test for Nicotine Dependence [FTND or “*FTCD*” hereafter, due to a suggested change of the original name to “*Fagerström Test for Cigarette Dependence (FTCD)*” to accurately describe the measured constructs]) [38, 39], PA engagement (Active Australia Survey [AAS]) [40], perceived benefits and barriers to PA (Exercise Benefits and Barriers Scales [EBBS]) [41], psychological distress (Kessler Psychological Discress Scale [K10]) [42], and acceptability of PA interventions (Treatment Acceptability and Preferences [TAP]) [43]. In the study context, alcohol and tobacco were considered to be legal substances; recreational (non-prescribed) cannabis use was classified as illicit according to current Australian law. If young people reported participating in a low number of times/minutes of PA per week (e.g., 60 min or less of physical activity per week, including active transportation and/or housework), this was referred to as “low PA engagement”. As part of the TAP, a measure which allows flexible integration of different descriptions of interventions, physical activity interventions were described as physical activity programs, adjunct to regular treatment, which could be tailored to a young person’s fitness level and preferences (e.g., length, intensity, supervision, indoor/outdoor setting). Based on this description, young people’s perceived efficacy, acceptability and suitability of the described intervention as part of substance use treatment services, as well as their willingness to comply with the described intervention, were assessed using a 4-point Likert scale. Five additional items were added to the EBBS questionnaire to assess young people’s views about associations between physical activity and substance use (e.g., “*Doing physical activity decreases my substance use”*).

The focus group followed a semi-structured format and was facilitated by one researcher and one silent moderator for a duration of 120 min; predefined questions were informed by the survey results and aimed to provide meaningful context to young people’s responses in the survey. Open-formatted questions probed the acceptability of PA interventions, perceived barriers and benefits to PA engagement and PA interventions, and preferences and needs regarding PA interventions and their integration into substance use treatment practice. The focus group was conducted according to an established framework of participatory design [44], which highlights the employment of creative, qualitative methods such as brainstorming as part of focus groups to help young people communicate [45]. It was further facilitated using Miro, an online whiteboard technology (https://miro.com/online-whiteboard/) and was recorded and transcribed verbatim using audio and video recording. Creative output (e.g., brainstorming) was captured using Miro.

## Data analysis

### Quantitative data analysis

IBM SPSS and the R programming language were used to explore study aim one - acceptability of PA interventions, PA engagement, psychological distress, substance use and perceived PA barriers in young people with problematic substance. SPSS, version 29.0.0.0 (IBM, New York, United States) was used for data preparation and cleaning, data corrections and manipulations and simple data explorations. Due to the violation of several parametric assumptions, including the skewness of the data, violation of normality and homogeneity of variances, robust methods and non-parametric approaches were applied. Missing value analysis (via the expectation maximisation (EM) method and Little’s MCAR Test) was not significant with X^2^ = 561.27 (590), *p* =.797, indicating that missing values were missing at random and no assumption was violated. Only 3.8% of all data (i.e. below 5%) were missing; missing values were excluded pairwise for each analysis. Based on the skewed data, variable relationships were explored using bootstrapped (10,000 resamples), two-tailed Spearman’s rank order correlations (rho); confidence intervals were calculated using the bias-corrected accelerated method (BCa). Group differences were investigated using Mann-Whitney U tests, Kruskal-Wallis tests or Fisher’s exact probability tests, with findings statistically significant at *p*≤.05. Data transformation (chi-squared and log-transformations) did not lead to more normally distributed data; thus, using robust analysis methods was deemed more appropriate [46, 47].

R Version 4.2.1 (GUI 1.79 High Sierra build, R Foundation for Statistical Computing, Vienna, Austria) was used for data mining and modelling, latent class and decision tree analyses. Regression tree analyses explored the explanatory power of relationships among participant characteristics within a model investigating treatment acceptability. Latent class analysis explored latent groups within manifested categorical data with expectation maximisation for model estimation [48]. Model fit was evaluated using the Bayesian Information Criterion, Akaike Information Criterion, and likelihood Ratio chi-squared statistic. Generalised additive modelling (GAM) using the Restricted Maximum Likelihood Model (REML) facilitated flexible predictive modelling without underlying assumptions of linear relationships.

### Qualitative data analysis

Qualitative content analysis explored study aim two - young people’s acceptability of PA interventions, perceived PA barriers, preferences and service-related needs, i.e., equipment, assistances, changes, minor processes that are necessary for the service to accomplish its purpose, regarding PA interventions (see study aim 2). Codes were derived inductively from data and defined during the analysis [49]. Categories were predominantly deduced from existing questions; codes, however, were derived from condensed meaning units and compared to preliminary established categories. Established codes were used as a coding scheme for subsequent coding. Quality criteria were implemented, including “trustworthiness” [50], consistency, reliability and reproducibility by following a detailed codebook and coding scheme [51].

### Mixed methods analysis

Qualitative and quantitative findings were integrated to identify areas of potential intervention to increase PA engagement and reduce SU as per the third study aim, and develop recommendations to inform the integration of PA into substance use treatment for young people. Findings were integrated within the *Capability- Opportunity- Motivation-Behaviour* (COM-B) model of behaviour [31], which describes three necessary conditions to provoke volitional behaviour (B): capability (C), opportunity (O) and motivation (M) [52]. Suitable areas for clinical services to intervene were analysed in line with this model.

## Findings/results

**Fig. 1:**
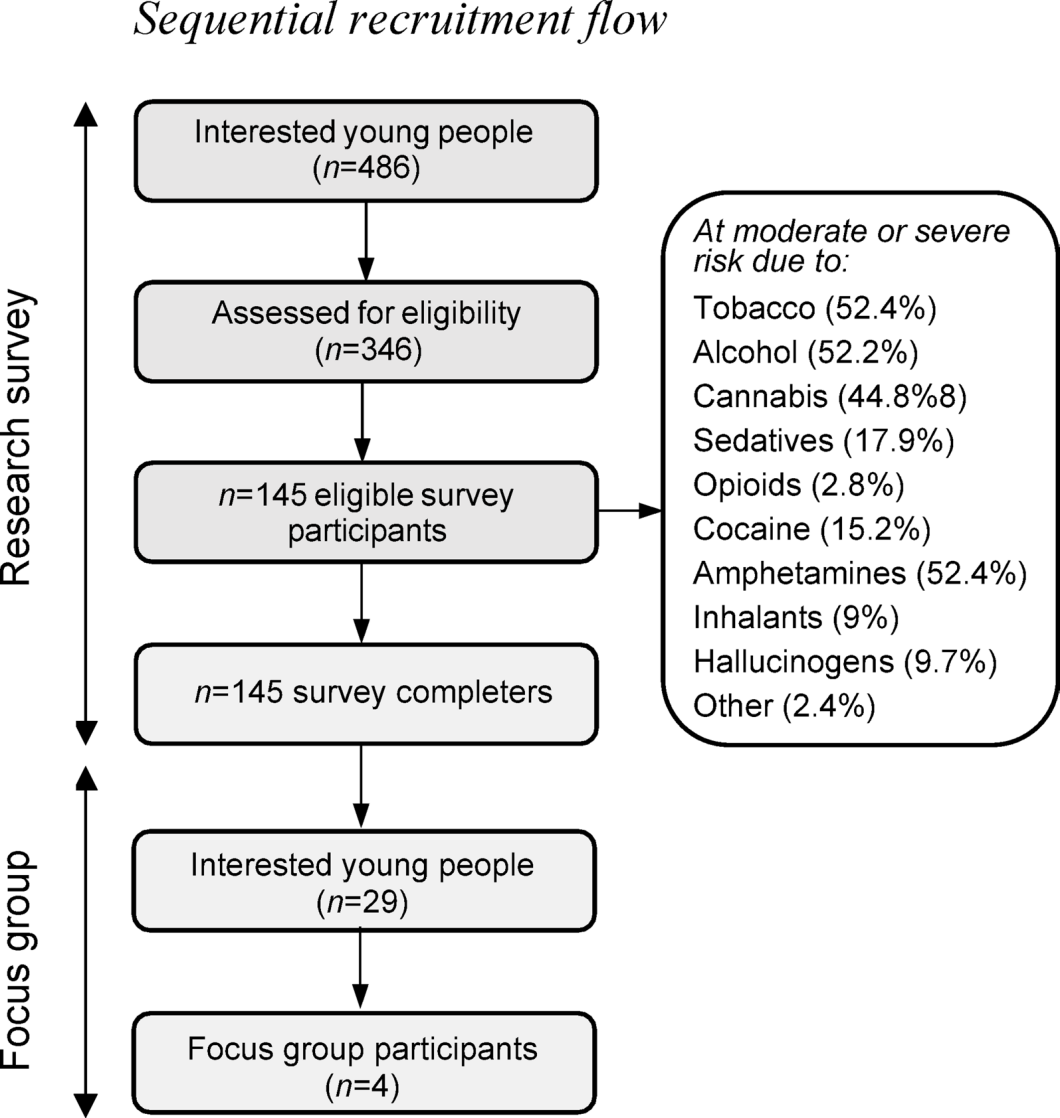
Sequential recruitment flow

### Quantitative findings

A total of 486 participants started the survey, 201 were ineligible after completing the screening questions, and *N* = 145 young people completed the survey. For recruitment flow, see Fig. 1.

The mean age was 21.9 (*SD* = 2.1) years. Participants predominantly identified as female (71.5%). No significant quantitative association was found for acceptability of PA interventions as part of SU treatment and severity/type of substance use. For additional characteristics and relationship analyses, see Tables 2 and 3.

**Table 2:** Sample characteristics

Characteristic	% (*n*)	M [95% CI]
**Substance-related risk** (as defined by the WHO ASSIST)		
Moderate risk	77.2% (112)	
High risk	22.8% (33)	
Only legal SU	16.6% (24)	
Legal & illicit SU	83.5% (121)	
Single substance use (lifetime)	14.5% (21)	
Polysubstance use (lifetime)	85.5% (124)	
**PA engagement** (as measured with the AAS)		
Walking min/week		315.1 min [239.89, 396.76]
Moderate PA min/week		78.0 min [39.55, 134.8]
Vigorous PA min/week		120.0 min [87.83, 149.86]
Total PA min/week		577.7 min [493.93, 823.48]
Sufficient PA	74/51%	
Insufficient PA	28/19.3%	
Not reported	43/29.7%	

Sufficient PA engagement calculated as recommended by the Active Australia Survey (AAS)

**Table 3:** Relationship analyses

Variable		PA min/week		PA times/week		Pychological distress	Treatment acceptability		
PA barriers		*r*= −0.33**		*r*= −0.42**		*r* = 0.44**		*r*= −0.3**	
PA benefits		*r* = 0.14		*r* = 0.22*		*r*= −0.32**		*r* = 0.55**	
Psychological distress		*r*= −0.15		*r*= −0.22*		—		*r*= −0.09	

Increased K10 (psychological distress) scores indicate higher levels of distress

Measurement tools: AAS: PA min/times per week; K10: Psychological distress; TAP: Treatment acceptability; EBBS: PA barriers/benefits

*p <0.05. **p <0.01

### Generalised additive modelling

The variable *PA barriers* significantly (*p* ≤0.001) predicted *treatment acceptability* via a mildly nonlinear smooth function, indicating that treatment acceptability decreases with increasing barrier perception. The low amount of explained variance (12.6%) and mildly nonlinear nature of the function indicated the presence of additional, unknown predictors or moderating variables.

**Fig. 2:**
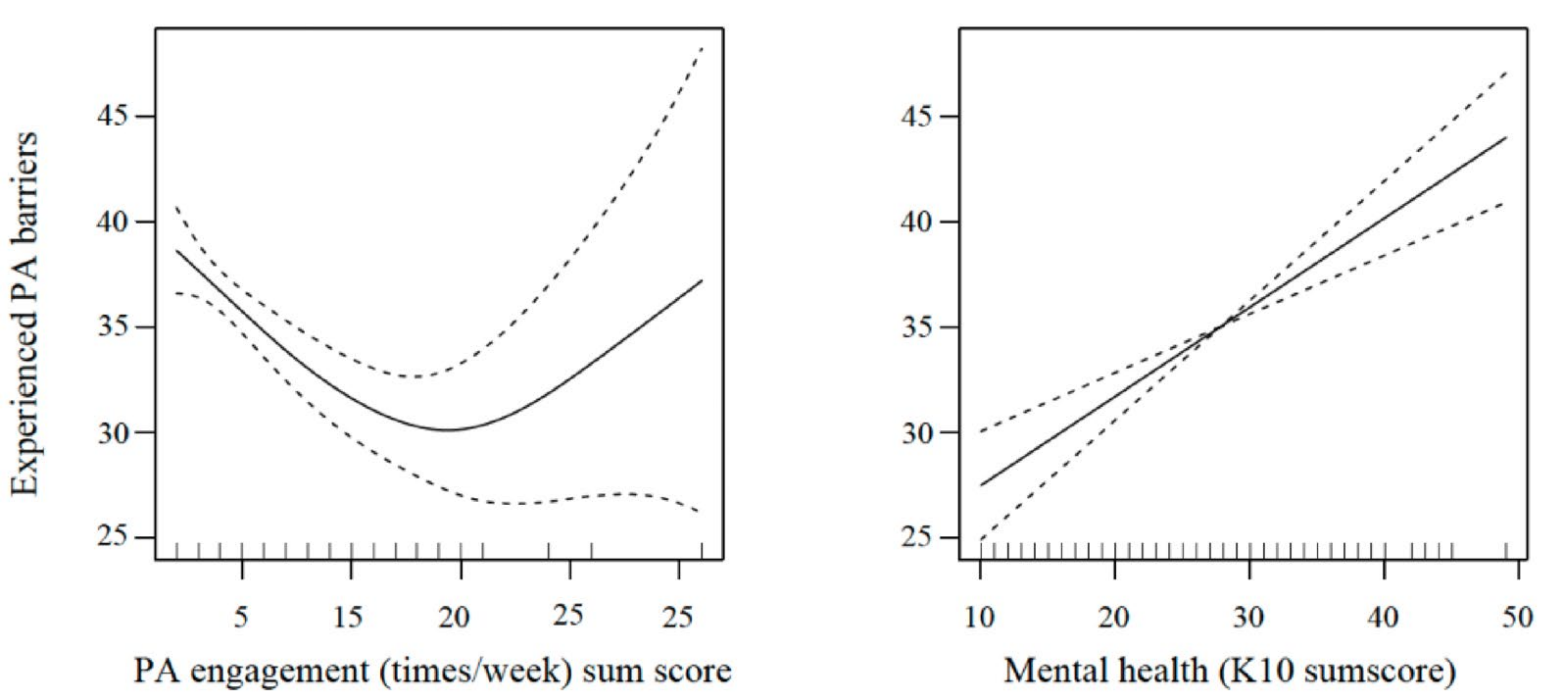
GAM of experienced PA barriers as predicted by features of mental health and PA participation (times/week)

*Psychological distress* (*p* ≤0.001) and the *frequency of PA engagement* per week (in times/week) (*p* ≤0.001) were predictors of perceived *PA barriers* to physical activity (see Fig. 2). The relationship between psychological distress and perceived barriers was linear (*edf* = 1). The relationship between PA times/week and perceived barriers, however, was nonlinear (*edf* = 2.55); indicating that a low frequency of PA engagement per week initially predicts a high amount of perceived PA barriers among young people, however, this relationship Changes once young people report participating in a high frequency of PA engagement per week. This could be explained by the presence of other mediating variables influencing the relationship, which appear only when participating in PA at least 25 times/week (Fig. 2). The combined explained overall variance of 36.7% indeed indicated the presence of other predictor variables.

### Latent class analysis

The latent class analysis (assessed for up to 10 classes) revealed a good fit for a 2-class model with a global maximum log-likelihood of −394.2. The model was based on the binarized indicator variables: Severe psychological distress (yes/no, cut-scores of K10), sufficiently active (yes/no, cut-scores of AAS), perceived PA barriers (low/high, cut scores of EBBS), treatment acceptability (low/high, cut scores of TAP) and overall substance risk level (moderate/high, cut-scores of ASSIST). Participants in latent class 1 were likely to report less psychological distress (74.2% chance), fewer perceived PA barriers (91.6% chance), higher treatment acceptability (61.2% chance) and sufficient PA levels (67.1% chance), and experience moderate substance-related risk (96.1%). Latent class 2 was likely to experience higher psychological distress (78.8%), insufficient PA levels (67.1%), and low PA treatment acceptability (56.6%).

### Regression tree analysis

**Fig. 3:**
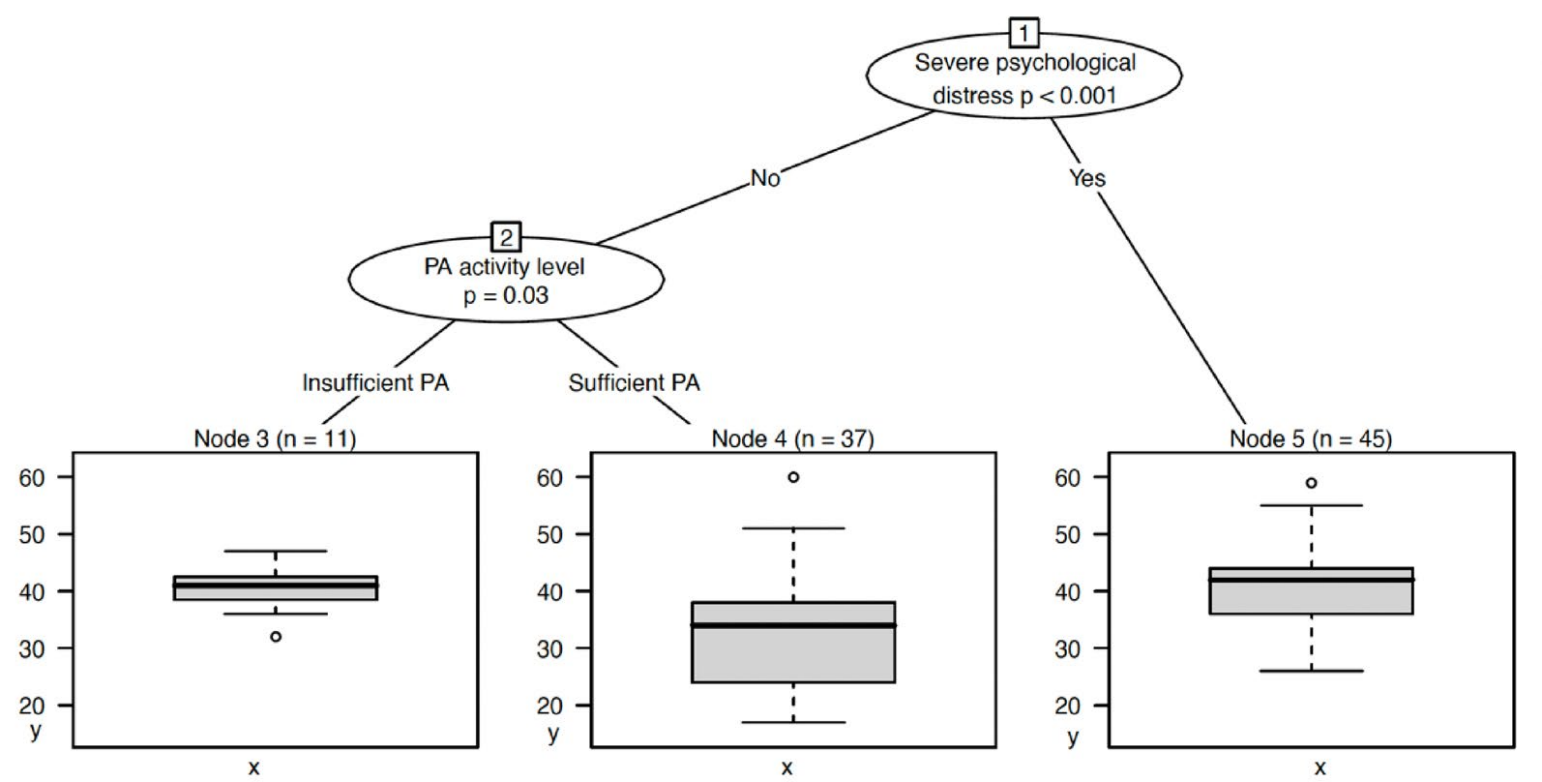
Conditional inference (“decision tree”) with indicated severe mental distress (as per K10) and PA activity level as independent variables (x-axis) and perceived PA barriers as dependent variable (y-axis). Decision points are referred to as “nodes”

Perceived barriers to PA were used as an indirect indicator of treatment acceptability. Psychological distress and PA activity levels were the strongest predictors of PA barriers (Fig. 3), indicating that young people with low psychological distress who engaged in sufficient PA reported the lowest mean value of PA barriers (*M* = 32.7, Node 4). Young people with severe psychological distress recorded the highest level of PA barriers (*M* = 41.5).

### Qualitative findings

A total of 29 young people initially indicated their interest in participating in the subsequent focus group. However, consistent with evidence about difficulties recruiting and retaining youth with SUDs into research studies, due to the commonly experienced complex challenges [53], 25 young people were lost to follow-up. Four young people (3 male, 1 female) participated in the focus group our young people (3 male, 1 female) participated in the focus group.

Seventy-seven distinct codes across five broad themes were extracted from written and spoken data. Each theme incorporated several categories.

### Benefits of PA and PA interventions

Reported benefits of PA included mental health benefits (e.g., empowering feelings), motivational benefits (e.g., increased motivation to quit substance use), benefits to life structure (e.g., routine), physiological benefits (e.g., ‘dopamine’ and energy increase) and a ‘behavioural domino effect’ (e.g., an increase in PA behaviour leads to an increase in other healthy behaviours).

Perceived benefits of PA interventions included substance use benefits (e.g., substance use reduction) and life structure (e.g., improved direction in life).

### Barriers to PA and PA interventions

Perceived barriers to PA interventions included logistical barriers (i.e., transportation barriers, lack of resources to reach clinical services or proximity barriers) and access barriers. Barriers to PA engagement were grouped into 4 categories: service-induced barriers (i.e., barriers caused by clinical services themselves), substance use barriers, social barriers, and “other” barriers. The most cited barrier to PA engagement was substance use (including drug “comedown”), which affected motivation to exercise, sleep, energy, fitness, and financial resources (e.g., to purchase gym membership or exercise equipment).

### Young people’s preferences regarding PA interventions

Young people’s preferences were grouped into three categories: clinician preferences, intervention preferences, and facilitation preferences. Young people preferred passionate clinicians knowledgeable in PA facilitation, able to provide clear directions as to where, how, how long, and at what intensity young people would perform physical activity. One young person explained: *“I think the biggest thing is whether they seem passionate about physical activity and can make you*,* um*,* want to engage. I think the title [of their profession] doesn’t really matter that much. It’s more whether they can make it engaging and show that they care about physical activity as well.”*

Preferred interventions were highly tailored and individually facilitated rather than group-based. Supervised physical activity was favoured in early treatment stages when habits had not yet been established; planned, unsupervised physical activity was preferred in later stages.

### Young people’s self-identified service-related needs regarding integrated PA interventions

Three categories of service-related needs were identified to increase participation in PA and PA interventions and reduce PA barriers: Service-led barrier reduction, service processes, and service provision. Active, service-led barrier reduction was believed to increase motivation, the value of PA engagement, positive feelings (e.g., less worry) and feelings of appreciation. Young people’s needs regarding service processes included increased progress checking, increased choice and improved information sharing among the treatment team.

**Fig. 4:**
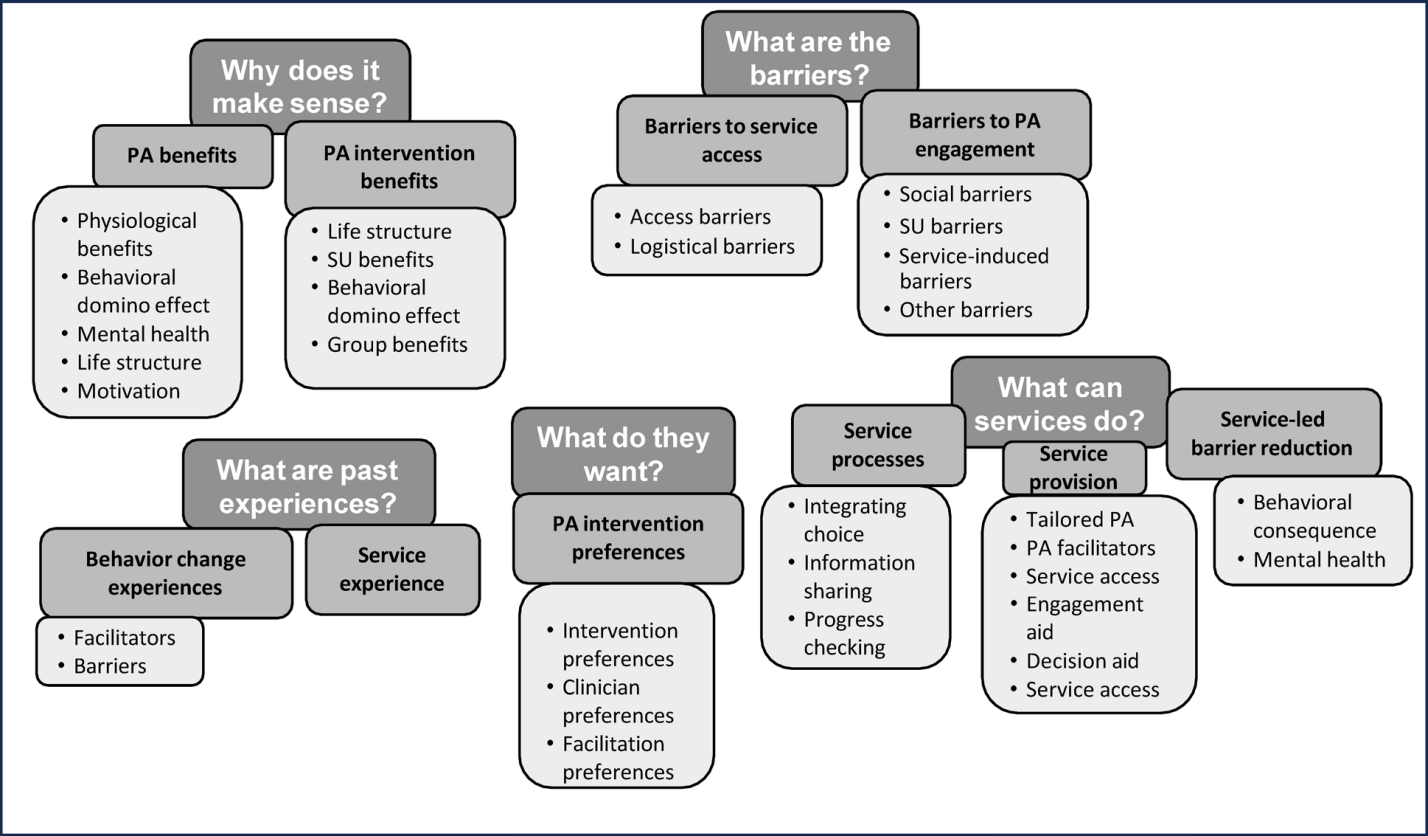
Themes, categories, and subcategories of young people’s perspectives on PA interventions. SU=Substance use; PA=Physical activity

Identified needs within the category or service provision included better access using local collaboration (e.g., collaboration between clinical services and schools or workplaces), organisational support (e.g., help with scheduling appointments), and improved access by proximity (i.e., reduced distance to clinical services). Engagement aids were also suggested, including reward systems for successful progress. Additional facilitators to help young people with problematic SU to engage in PA overall included additional psychological support, low-cost physical activity, exercise variety, scheduling support and learning more about the benefits of PA overall. Identified themes, categories and sub-categories are summarised in Fig. 4.

#### Mixed methods findings: interpretative discussion

Quantitative and qualitative findings indicate a high rate of treatment acceptability of physical activity interventions among young people with problematic substance use and a strong willingness to engage in such interventions. Similar overarching themes were identified in both datasets in the reported benefits of PA, including mental health benefits (e.g., decreased stress, improved mood), behavioural benefits (e.g., improved functioning, increased health behaviours), and social benefits (e.g., social encouragement and connection). Some differences between the datasets exist: Physiological benefits of PA participation (e.g., improved fitness and stamina) were identified only in the quantitative dataset, benefits of PA participation for life structure (e.g., routine) were identified only in the qualitative dataset. The difference may be explained by the flexible nature of the focus group, which facilitated a broader exploration of themes compared to the research survey.

The association of mental health and physical activity in young people with problematic substance use was addressed in both datasets. Young people qualitatively reported a reduction of psychological distress through PA participation; quantitative results indicated a significant association of PA participation, psychological distress and perceived barriers to PA engagement. This highlights the bidirectional relationship between mental health and PA engagement [54]; that is, the importance of addressing mental health concerns to increase the likelihood of participating in PA interventions, but also the potential to improve young people’s mental health through targeted, achievable PA plans.

For perceived barriers to PA engagement, quantitative results predominantly indicated time and physical exertion to be overarching barriers, while qualitative results particularly highlighted substance use-related barriers to PA and barriers relating to clinical services (e.g., vague PA recommendations provided by clinicians). Both datasets reported substantial social barriers to PA, including a lack of social support for PA engagement, cultural barriers, gender barriers, or negative group dynamics.

### Strategic alignment of service-related needs, barriers/benefits of PA onto COM-B

The reported barriers, benefits, and perceived service-related needs identified during quantitative and qualitative data collection were mapped onto the COM-B factors to investigate how young people’s experiences may impact behaviour change and identify potential target areas for interventions (see Table 4). This allowed an exploration of how the perceived barriers may affect the factors that are relevant to achieving behaviour change and how the benefits of PA and PA interventions may potentially improve the likelihood of behaviour change by positively impacting the opportunity, capability, and motivation of PA engagement of young people.

**Table 4:** Integration of young people’s perspectives using the COM-B model

COM-B dimensions	Construct descriptions [55]	PA/PA intervention benefits	Barriers to PA/PA intervention	What clinical services can do (“referred to as service-related needs”)
Capability	The individual has interpersonal competence, knowledge, and skills to fulfil the role.	*Physical capability*: - Increased physical health/wellbeing/energy - Increased stamina/fitness - Improved sleep *Social capability*: - Increased social skills *Psychological capability*: - Knowledge on PA benefits - Improved mental health/reduced psychological distress - Empowerment - Increased self-efficacy	*Physical capability*: - Tiring - Fatiguing *Social capability*: - SU related barriers *Psychological capability*: - Psychological distress - Limited knowledge on PA and substance use - SU related barriers	- Tailored PA - Behaviour change interventions - Informative education on PA and SU - Continuous progress checking - Decision aids - Engagement aids - Combination of psychological and PA support - Supervision according to treatment stage
Opportunity	The individual has availability, scope, and power to fulfil the role. [i.e., factors that lie outside of an individual; 31]	- Life structure - Increased social connection (social opportunity)	- Limited access - Limited financial resources/equipment etc. - Limited social opportunity (cultural restrictions, social stigma) - Not enough time - Logistical barriers - Service-induced barriers	- Increased service access - Local connection - Service-led barrier reduction
Motivation	The individual is committed to fulfilling the role.	- Habit forming - Enjoyment - Entertainment - Motivation increase	- Lack of motivation - Substance use (affects motivation) - Partner/family discouragement - Hard work	- Motivating/passionate clinician - Integrating choice

Table 4 highlights potential areas where clinical services could intervene and potentially improve the capability, opportunity and motivation of young people with problematic substance use to increase PA participation. In line with the model’s theory, improvement of all three factors through either barrier reduction or increase in benefits leads to behaviour change, i.e., an increase in PA engagement, which may reduce substance use. Behaviour change (i.e. increased PA activity) affects and improves the three COM-B factors through positive feedback loops.

## Discussion

This mixed-methods study investigated young people’s perspectives regarding the integration of PA interventions into treatment practice. Study aim 1 explored acceptability of PA interventions (predictors and correlates), PA engagement, mental health and perceived PA barriers in young people. Quantitative findings reported high acceptability of PA interventions and a significant positive relationship between psychological distress, physical activity engagement and treatment acceptability. GAM confirmed PA barriers to be a significant predictor of treatment acceptability, while psychological distress and PA engagement predicted barrier perception. Regression tree analyses highlighted the importance of PA activity levels and psychological distress for the perception of PA barriers.

According to study aim 2, which examined young people’s preferences and insights regarding the integration of PA interventions, qualitative findings highlighted the importance of addressing mental health concerns, loneliness, and social connection as part of PA interventions. Perceived barriers to PA included substance use, which highlights the interaction of substance use and physical activity from a qualitative perspective. Identified intervention preferences included planned, individual PA sessions, with different supervision formats being favoured in different treatment stages.

Integration of perceived barriers, benefits, facilitators and needs of young people within the COM-B model of behaviour Change was conducted to explore study aim 3, which investigated areas of potential intervention to increase PA engagement in young people. Findings highlighted how active reduction of perceived barriers and increased perception of PA benefits may increase young people’s opportunity, capability and motivation to engage in PA and PA interventions.

### Previous research findings

While there is only limited research investigating the acceptability of physical activity interventions as part of substance use treatment, the current study and previous research report overall high acceptability [56]. One previous study reported high acceptability of a PA intervention for cannabis reduction in young people [57].

Barriers to PA in existing literature are rarely reported for young people with problematic substance use, but only for young people overall. Caution thus needs to be applied with consideration of the unique barriers that may apply to young people with problematic substance use but not to other young people. A recent review investigated barriers to PA interventions in young adults and adults who use substances [25]. The review highlighted similar barriers to the current study, including physical fitness, substance-related barriers, perceptions and judgments of others, low motivation, logistical factors (e.g., access and affordability), and time factors, including competing responsibilities. Overlapping service-related needs included the importance of applying flexible intervention formats according to each person’s progress through treatment, for instance, group-based intervention in early treatment stages, and individual interventions in later treatment stages.

### Strengths and limitations

This study has several strengths. To our knowledge, it was the first to investigate treatment acceptability of PA interventions and correlates thereof in young people with problematic substance use, as well as PA barriers perceived by affected young people. Findings may shed light on known treatment challenges within this cohort, e.g., low motivation, by increasing understanding of PA barriers that these young people experience. The mixed methods approach facilitated an in-depth exploration of young people’s perspectives on the integration of tailored PA interventions into substance use treatment practice. It further highlights the value in gaining young people’s insights on their unique experiences with problematic substance use and its impact on their lives, and recognises the challenges and service-related needs of this population, which may differ from other young people. The study allowed young people to participate in a meaningful way and contribute to research which will inform future PA intervention development for this population.

Some limitations exist. Recruitment through university services and social media platforms may have led to selection bias in the accurate representation of different socioeconomic positions. Further, the self-selection of participants into the study may have led to bias and inaccuracies within the study results. For instance, self-selection into the study possibly leads to inflated levels of treatment acceptability for PA interventions [58]. The risk of social desirability bias was judged minimal due to the anonymous nature of the online survey and the exclusion of known themes with the potential to trigger socially desirable response patterns from the focus group [59]. According to eligibility criteria, young people were included if they reported current and/or past engagement with clinical services, and those who indicated willingness to seek services. Findings may have been different if solely young people currently engaged with clinical services were included. The quantitative data presented numerous outliers and highly skewed distributions, which are common for substance-use populations [60, 61]. The English-speaking participant group and lack of ethnic diversity may limit generalisability to other populations of young people. Another limitation was the size of the focus group, despite recommendation [36], for achieving data saturation. Last, the use of self-report measures has been widely discussed in relation to validity [62], with arguments speaking both for and against the use of self-reports [63–65]. Given potential limitations of self-report data, findings should be interpreted with caution.

### Implications and recommendations

Findings shed light on the acceptability of PA interventions as part of substance use treatment and may support clinicians’ decisions to integrate PA into a young person’s treatment plan. Findings show that young people value the role of PA as part of treatment due to the many perceived benefits. However, several factors are outlined which need to be considered when integrating PA interventions into substance use treatment practice, i.e. the complex needs, perceived barriers, and symptom presentation of young people with problematic substance use.

Findings on latent classes among young people and the decision tree analysis may inform a streamlined decision-making process from a clinician’s perspective regarding a young person’s capability, i.e., psychological and physical capacity, to engage with a PA intervention integrated with their regular treatment according to the COM-B behaviour model [31]. Classification of incoming clients according to their symptom presentation in line with the latent class analysis or decision tree analysis may inform clinicians’ decision-making regarding the sequence in which complex needs are addressed, e.g., which clients may be able to participate in a PA intervention and which clients may benefit from a reduction of psychological distress first.

This study further demonstrates that young people perceive problematic substance use to affect their PA engagement, motivation, sleep, energy, fitness, and financial resources. This suggests that young people with problematic substance use may benefit from receiving additional support regarding their engagement in both regular PA and PA interventions if offered by clinical services. The large number of identified mental health and social barriers to PA engagement indicates that the integration of PA interventions with mental health support and social support should also be considered.

Several preferences and service-related needs were highlighted; consideration of these needs as part of PA intervention development may increase engagement and motivation in young people and improve the provider-client relationship overall. Further, providing youth-centred care, giving young people choices in the selection of activities/exercises, and allowing them to contribute to their own treatment decisions may increase their feelings of being valued, leading to a better appreciation of clinical services in return.

The integration of quantitative findings into the COM-B model of behaviour outlines the complex interaction of PA barriers, benefits and service-related needs of young people on different dimensions (i.e., capability, motivation, opportunity). As such, the model may inform clinical services’ intervention strategies through consideration of the bidirectional relationships between factors that affect mental health, physical activity behaviour and substance use.

### Directions for future research

Overall, more research which investigates PA interventions for problematic substance use in young people is suggested, with consideration of the unique needs, experiences, and perspectives of affected young people. Exploration of the perspectives of a larger and more ethnically diverse participant group (across genders) may increase the generalisability of findings. Last, young people’s participation and integration as equal partners in research, intervention design and development should be considered.

## Conclusion

Physical activity interventions are perceived as highly acceptable and suitable for substance use reduction by young people with problematic SU aged 16–25 years. However, young people differ in their psychological distress (i.e., mental health) and PA levels among other factors, likely impacting PA barriers and treatment acceptability. Consequently, some young people may be more capable of participating in integrated PA intervention, while others may benefit from simple PA support first. This research may inform decision-making as to when and to whom to offer integrated PA intervention as part of treatment, and what the treatment sequence should be. It further gives insight into some of the unique barriers to PA young people with problematic substance use experience; their preferences and service-related needs, in turn, should be considered as part of intervention development and implementation.

## Data Availability

All data and research materials are available upon reasonable request to the corresponding author.

## Abbreviations

AAS: Active Australia Survey
ASSIST: Alcohol, Smoking, and Substance Involvement Screening Test
COM-B model: Capability Opportunity Motivation Behaviour model
EBBS: Exercise benefits & Barriers Scales
K10: Kessler Psychological Distress Scale
PA: Physical activity
SU: Substance use
TAP: Treatment Acceptability and Preferences Measure
WHO: World Health Organization
